# Potentially inappropriate prescribing (PIP) in older people and its association with socioeconomic deprivation—a systematic review and narrative synthesis

**DOI:** 10.1186/s12877-024-04858-w

**Published:** 2024-08-02

**Authors:** Adrian James Hire, Bryony Dean Franklin

**Affiliations:** 1 Centre for Medication Safety and Service Quality, Imperial College Healthcare NHS Trust, London, UK; 2 UCL School of Pharmacy, London, UK

**Keywords:** Potentially inappropriate prescribing, Older people, Socioeconomic status, Systematic review

## Abstract

**Background:**

Potentially inappropriate prescribing (PIP) refers to the prescription of medications that carry a higher risk of adverse outcomes, such as drug interactions, falls, and cognitive impairment. PIP is of particular concern in older adults, and is associated with increased morbidity, mortality, and healthcare costs. Socioeconomic deprivation has been identified as a potential risk factor for PIP. However, the extent of this relationship remains unclear. This review aimed to synthesize the current literature on the association between PIP and socioeconomic status (SES) in older adults.

**Methods:**

A literature search was conducted using the databases Medline, Embase and CINAHL. A search strategy was developed to capture papers examining three key concepts: PIP, socioeconomic deprivation and older/elderly populations. Peer-reviewed quantitative research published between 1/1/2000 and 31/12/2022 was eligible for inclusion.

**Results:**

Twenty articles from 3,966 hits met the inclusion criteria. The sample size of included studies ranged from 668 to 16.5million individuals, with the majority from Europe (*n* = 8) and North America (*n* = 8). Most defined older patients as being 65 or over (*n* = 12) and used income (*n* = 7) or subsidy eligibility (*n* = 5) to assess SES. In all, twelve studies reported a statistically significant association between socioeconomic deprivation and an increased likelihood of experiencing PIP. Several of these reported some association after adjusting for number of drugs taken, or the presence of polypharmacy. The underlying reasons for the association are unclear, although one study found that the association between deprivation and higher PIP prevalence could not be explained by poorer access to healthcare facilities or practitioners.

**Conclusion:**

The findings suggest some association between an older person’s SES and their likelihood of being exposed to PIP. SES appears to be one of several factors that act independently and in concert to influence an older person’s likelihood of experiencing PIP. This review highlights that prioritising older people living in socioeconomically-deprived circumstances may be an efficient strategy when carrying out medication reviews.

**Supplementary Information:**

The online version contains supplementary material available at 10.1186/s12877-024-04858-w.

## Introduction

The ageing of the global population has been described as ‘one of the most important economic, social, and medical issues of current times’ [1]. Although the exact definition of what constitutes an older person varies [2], both developed and developing countries are facing ‘an unprecedented and rapid rise in the number of elderly people’ [3]. Against a background of falling birth rates, the global population aged 60 years and older has overtaken the number of children aged under 5 years; by 2050 older people are projected to comprise 22% of the population (equivalent to 2.1 billion individuals) [4]. Living longer is not an issue in itself. However, ageing is a well-established risk factor for a number of noncommunicable diseases, including cardiovascular disease, stroke, various cancers, osteoporosis and dementia [5]. With individuals living longer lives, more people are developing these diseases of ageing. Multimorbidity, or the presence of multiple long-term conditions, is very common among older adults, with its prevalence increasing with age [6]. One meta-analysis of 193 international studies estimated that 47.6% of adults aged between 59 and 73 years of age had multiple long-term conditions, with this figure rising to 67.0% among adults aged 74 years or older [7].

Prescribed medication plays an important role in the management of long-term conditions. Although beneficial in many cases, the use of prescribed medications in older people is not without risk. In an attempt to prevent or treat multiple conditions, potentially inappropriate prescribing (PIP) can occur. PIP may refer to the prescription of potentially inappropriate medications (PIM) associated with a higher risk of adverse outcomes such as drug interactions, falls, and cognitive impairment [8]; it can also encompass the omission of potentially beneficial medications and the use of appropriate medications at inappropriate doses, or for inappropriate lengths of time [9]. However it is defined, PIP is an established cause of morbidity, mortality, and increased healthcare costs [10].

Socioeconomic deprivation is another factor that can affect health [11]. In the case of older people, exposure to deprivation can augment the negative health effects of ageing [12]. People living in more socioeconomically-deprived circumstances tend to report poorer health, and have a higher likelihood of developing multiple long-term conditions [13]. Additionally, it has been hypothesised that persons living in deprived areas may have less access to high-quality health and care services, despite having a relatively increased need for such provisions [14]. What is less clear is whether older people living in deprived areas are also more likely to experience PIP.

This systematic review aims to synthesize the current literature on the association between PIP and socioeconomic deprivation in older adults. By examining the available evidence, we aim to provide insight into the relationship between PIP and socioeconomic deprivation and identify potential areas for intervention.

## Methods

### Data sources

A systematic search was conducted using Medline (via OVID), Embase (via OVID) and the Cumulative Index to Nursing and Allied Health Literature (CINAHL). Peer-reviewed quantitative research papers published between 1 January 2000 and 31 December 2022 were eligible for inclusion, with this period chosen to reflect contemporary practice. Reviews and commentaries were excluded. No language or geographical restrictions were applied. A search strategy was developed to capture papers examining three key concepts: (1) PIP, (2) socioeconomic status and (3) older/elderly populations. The full search strategy can be found in Supplementary Material 1.

### Study selection

To be included, studies had to meet each of the following criteria:

Population: Studies examining older people, either as a standalone population or as a defined subgroup within a broader population. Although a threshold of 60 [15] or 65 years [16, 17] of age is often used, there is no universally agreed definition of what constitutes an ‘older person’ [2]. Due to international (and national) variations in average lifespans, and an increasing mean age in many countries, a definition of an ‘older person’ in one context may not be appropriate in another. As such, papers referring to older, elderly or geriatric patients were included, regardless of the age criterion used.

Exposure: Socioeconomic deprivation. A number of distinct measures of socioeconomic status (SES) were deemed appropriate for the assessment of deprivation:

- Studies referring to income and wealth-based measures of SES in their title or abstract were included. Income has been described as ‘the best single indicator of material living standards’ [18], although wealth (reflecting both financial and physical assets) may be more important in older age due to the accumulation of wealth over time, and the impact of retirement on income [19]. Both absolute and relative measures of income and wealth were deemed relevant. For example, a study examining PIP among individuals at different income deciles would be included, as would a study looking at persons with a level of income above or below the poverty threshold of a particular country. Collectively, such studies were termed as using ‘income based measures’ of SES. Studies that assessed deprivation status based on reported ability to afford resources, or based on the possession of certain assets were termed as using ‘self-reported’ measures of SES.
- Studies that measured SES using a deprivation score or index such as the Index of Multiple Deprivation (IMD) [20] were also included, if the index used featured some income-based component. These were referred to as ‘composite measures’ of deprivation.
- A person’s eligibility for income-dependent governmental programs, such as Medicaid in the US, was also deemed an acceptable measure of SES [21]. Such papers were defined as using ‘subsidy eligibility’ to assess socioeconomic deprivation.
- Finally, studies making international comparisons in PIP incidence were also eligible, if an appropriate income or wealth-based measure was used to compare the countries analysed (for example, gross domestic product [GDP] per capita). These were defined as ‘international measures’ of socioeconomic deprivation.

Outcome: Studies examining PIP. This could be a reference to PIP as a concept, or a single, specific example of PIP (for example, the use of warfarin in individuals with active gastrointestinal bleed). PIP was taken to include:

- Treatment with medications that have an *unfavourable risk–benefit balance* for a given patient. These may be defined as potentially inappropriate medications (PIM).
- Treatment with medications or formulations that are *less preferred* due to the availability of safer prescribing options.
- Treatment with medications at *inappropriate doses* (either too high or too low for a given patient).
- Treatment with medications for an *inappropriate length of time* (either too long or too short).
- Treatment with medications that *interact with concomitant medications or disease states* in such a way that has the potential to cause harm.
- The *omission* of medications that would likely be of benefit for a given patient.

To be included, the appropriateness of prescribing could be assessed subjectively, or using objective criteria (eg STOPP/START criteria [22], Beers criteria [23]) or using prescribing guidance (eg the British National Formulary, or national prescribing guidelines). It was recognised that polypharmacy could be appropriate in some patients (for example, those with multiple long-term conditions) and inappropriate in other patients. As such, papers examining polypharmacy were included only if they explicitly examined *potentially inappropriate* polypharmacy.

### Study types, setting and reporting of results

All observational study types were eligible for inclusion, with no restrictions on setting. To be included, papers stating an association between SES and PIP had to report a measure of statistical significance underlying the result such as p-values and/or confidence intervals.

### Screening process

Citations identified via literature search were uploaded to Covidence, and both automated and manual de-duplication performed. Titles and abstracts were then screened by the lead author to identify papers appropriate for data extraction, with a second reviewer (SK) examining a 20% random sample. Cohen’s kappa was used to assess agreement. Following this, ‘full text’ versions of studies potentially meeting the review’s inclusion criteria were retrieved. These were then reviewed by the lead author, with the co-author (BDF) screening a 10% random sample to assess agreement. At this stage, any disagreements were resolved by discussion, and all papers selected for review were subject to data extraction and quality appraisal. Where full texts were unavailable in English, Google Translate was initially used assess whether the paper was likely to meet the inclusion criteria. Where potentially relevant information was identified, this was reviewed by a speaker fluent in that language to confirm that the inclusion criteria were met, and to assist with data extraction.

### Data extraction and quality appraisal

We extracted the following information using an Excel spreadsheet: title, first author, year of publication, country, setting and participants, measure/definition of deprivation used, measure/definition of PIP used, and relevant data extracted regarding the association between SES & PIP. Quality appraisal was carried out by the lead author using tools provided by the Joanna Briggs Institute (JBI), with the appropriate tool selected according to study type [24]. A second reviewer (CF) independently screened a sample of papers to assess agreement.

### Data synthesis and analysis

A narrative synthesis was used, as we anticipated that heterogeneity in contexts, definitions of older people, and the measures and definitions of socioeconomic deprivation used would preclude meta-analysis. The review was prospectively registered with the International Prospective Register of Systematic Reviews (PROSPERO; registration number CRD42023385451). The review follows the Preferred Reporting Items for Systematic Reviews and Meta Analyses (PRISMA) [25] and the Synthesis without meta-analysis (SWiM) reporting guidelines [26]. A completed PRISMA checklist can be found in Supplementary Material 2.

## Results

**Fig. 1:**
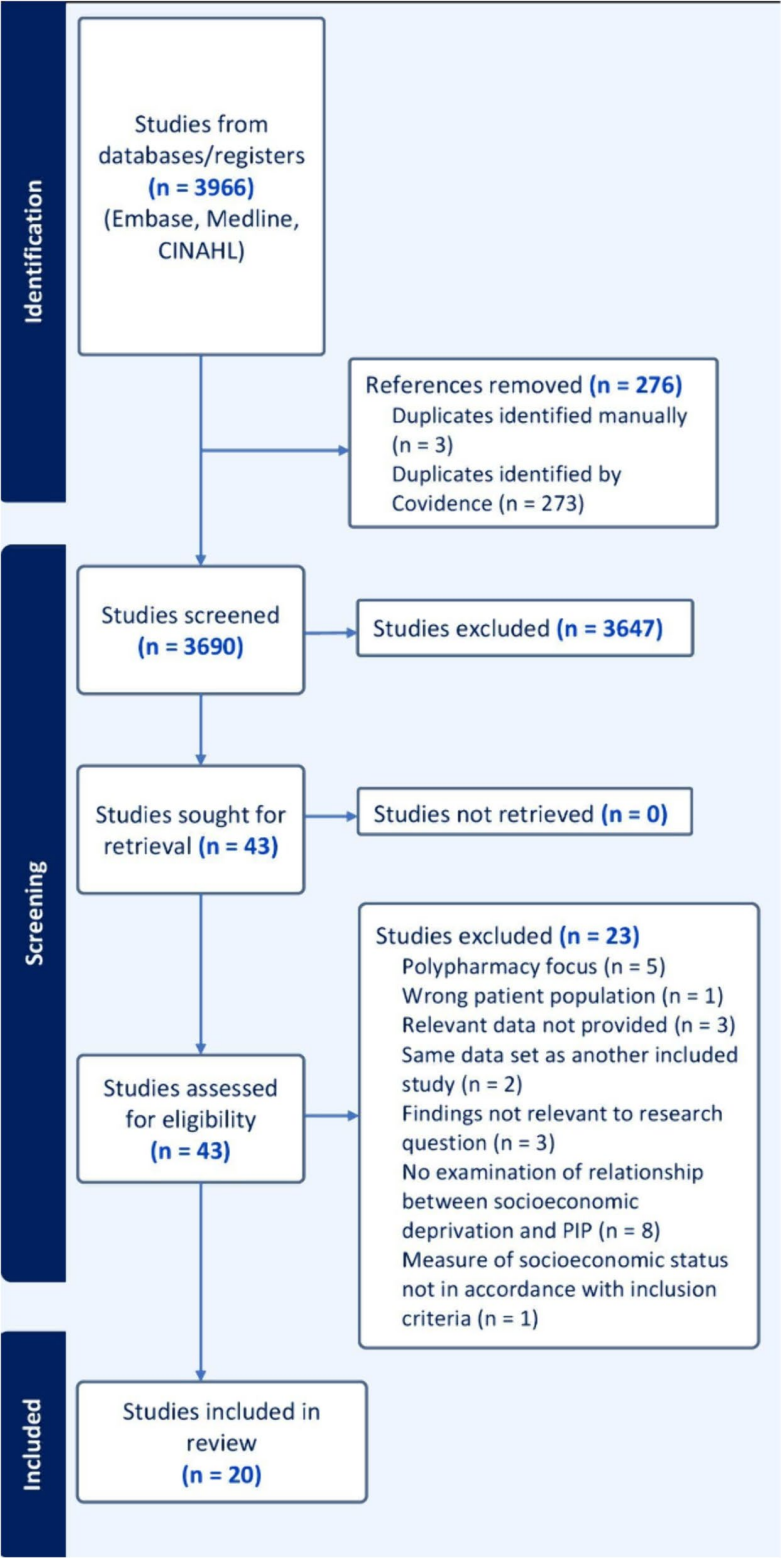
PRISMA diagram

A total of 3,966 references were identified, with 276 removed by a combination of automatic and manual deduplication. After screening for eligibility based on title and abstract, 43 advanced to full text screening. At this stage, a further 23 articles were excluded. In total, 20 papers were included in the final analysis (Fig. 1). A full summary of included papers can be found in Appendix 1, with a brief overview in Table 1. Identified studies generally focussed on older people aged 65 years or over (*n* = 18). Studies were from Europe (*n* = 8), North America (*n* = 8), South America (*n* = 2) and Asia (*n* = 1). A further publication [27] featured data from 37 countries. Most used multivariable analyses, examining a range of risk factors to assess their potential association with PIP, rather than focussing on socioeconomic factors alone. The sample size of included studies ranged from 668 individuals up to 16.5million.

**Table 1:** Overview of included studies (presented alphabetically according to first author)

First Author & Year (Country) (citation)	Findings regarding socioeconomic status (SES) and potentially inappropriate prescribing (PIP)^a^	Age of participants	Measures used to identify PIP^b^	Measure(s) of SES used	Measure of SES classification^c^	Quality score^d^
Abraham 2020 (USA) [28]	**Statistically significant association between SES and PIP**	65 +	Beers criteria 2015	Medicaid eligibility	Subsidy eligibility	8/8
Beuscart 2017 (France) [29]	**Statistically significant association between SES and PIP**	75 +	Laroche list	Municipality average taxable income, municipality average non-taxable income	Income	7/8
Blackwell 2012 (USA) [30]	**Statistically significant association between SES and PIP**	65 +	Beers criteria 2003	Medicare & Medicaid dual eligibility	Subsidy eligibility	8/8
Bongaerts 2021 (Muti-center) [27]	**Statistically significant association between SES and PIP**	65 +	HbA1c measurement, Initiation of medication(s) with a high risk of hypoglycemia	Gross national income of participants’ country of residence	International measure	8/8
Carey 2008 (UK) [31]	**Statistically significant association between SES and PIP**	65 +	Beers criteria 2003	Index of Multiple Deprivation score, Acorn index classification	Composite measure	7/8
Chauvin 2021 (Switzerland) [32]	*No significant association*	68 +	Laroche list	Receipt of income-related social support, self-reported assets higher than contemporaries	Multiple: Subsidy eligibility **and** self-reported economic situation	8/8
Coelho Filho 2004 (Brazil) [33]	**Statistically significant association between SES and PIP**	60 +	Beers (version not stated)	Area socioeconomic status, derived from Brazilian census data	Composite measure	7/8
Extavour 2018 (USA) [34]	*No significant association*	65 +	Beers criteria 2012/15	Median household income by zip code, Medicaid eligibility	Multiple: Income **and** subsidy eligibility	7/8
Fialova 2005 (Muti-center) [35]	**Statistically significant association between SES and PIP**	65 +	Adapted Beers criteria 1997/2003, McLeod criteria 1997	Self-reported economic situation	Self-reported economic situation	7/8
Holmes 2013 (USA) [8]	**Statistically significant association between SES and PIP**	65 +	Beers criteria 2003	Eligibility for low-income subsidy	Subsidy eligibility	8/8
Hwang 2023 (USA) [36]	Mixed findings	66–90	Beers criteria 2019	Income, Neighbourhood Area Deprivation Index, cumulative SES score	Multiple: Income **and** Composite measure	8/8
Hyttinen 2019 (Finland) [37]	Inverse association between socioeconomic deprivation and PIP	65 +	Meds75 + classification	Household spending money divided by number of persons living in the household	Income	7/8
Joung 2019 (South Korea) [38]	**Statistically significant association between SES and PIP**	70 +	Beers criteria 2015	Income	Income	8/8
Lechevalier-Michel 2005 (France) [39]	Mixed findings	65 +	Beers criteria, with adaptions to reflect French prescribing practice	Household income	Income	8/8
Lesen 2010 (Sweden) [40]	**Statistically significant association between SES and PIP**	75 +	Swedish National Board of Health and Welfare classification	Family disposable income divided by the square root of family members	Income	8/8
Lutz 2017 (Brazil) [41]	*No significant association*	60 +	Beers criteria 2012	Brazilian Association of Research Companies classification of economic level	Composite measure	8/8
Miller 2017 (USA) [42]	*No significant association*	65 +	Beers criteria 2012	Income as a percentage of federal poverty level, Medicaid eligibility	Multiple: Income **and** subsidy eligibility	7/8
Morgan 2016 (Canada) [43]	Mixed findings	65 +	Beers criteria 2012	Household income	Income	7/8
Odubanjo 2004 (Ireland) [44]	**Statistically significant association between SES and PIP**	70 +	Indicators determined by study authors, plus some Beers indicators	Point of enrolment in General Medical Services scheme	Subsidy eligibility	7/8
Rahman 2020 (USA) [45]	**Statistically significant association between SES and PIP**	65 +	Beers criteria 2015	Income	Income	8/8

^a^In the case of studies where alternative indicators of socioeconomic status were also provided (for example literacy, or rural living situation), these were not quoted in this review for reasons of consistency and clarity

^b^Studies used either all or just some of the indicators listed in the tool

^c^Classification used to group studies for analysis according to the type of SES measure used

^d^Joanna Briggs Institute (JBI) criteria

### Results of screening process and quality appraisal

For title and abstract screening, a Cohen’s kappa of 0.8 was obtained, indicating ‘substantial agreement’. All 20 included studies scored either 7 or 8 out of a possible 8 using the JBI critical appraisal checklist. A second reviewer (CF) independently screened three included studies; scores for two of the papers were identical, with a further study being scored 8 by the lead author and 7 by the second reviewer.

### Overview of findings

Study findings were mixed. Most multivariate studies reported a statistically significant but modest association between SES and PIP after adjusting for other factors (*n* = 12) [8, 27–31, 33, 35, 38, 40, 44, 45]. A further three had more mixed findings – for example, finding a significant relationship in certain subgroups only, or coming to different conclusions when different measures of SES were used [36, 39, 43]. For example, in Canada, Morgan and colleagues reported a significant relationship between SES and PIP in older males but not females [43]. In the United States, one study found that the socioeconomic status of an individual’s neighbourhood, but not their household income, appeared to significantly influence their chance of being prescribed two or more PIMs [36]. Of the remaining six studies identified, four concluded that there was no statistically significant link between SES and PIP [32, 34, 41, 42], and one Finnish study reported an inverse association between income and the use of PIMs [37].

### Findings by age threshold used

All four papers with minimum age thresholds of 70 and 75 years reported a significant association between deprivation and PIP incidence, despite using different PIP measures: the Laroche List [29], the Swedish National Board of Health and Welfare classification (NBHW) criteria [40], and two adaptions of the Beers criteria [38, 44]. However, the majority of included papers defined older patients as being at least 65 years of age (*n* = 12) [8, 27, 28, 30, 31, 34, 35, 37, 39, 42, 43, 45]. These gave a more mixed picture of the relationship between SES and PIP. Seven of the papers reported a significant association between deprivation and PIP [8, 27, 28, 30, 31, 35, 45], with another two giving mixed results [39, 43]. A further two focusing on the over 65s found no significant association between SES and PIP [34, 42], and one reported an inverse relationship between SES and the likelihood of being prescribed a PIM [37]. The two Brazilian studies used the lowest age threshold of the studies included, with both focusing on individuals aged 60 years and older [33, 41], and both using (different) composite measures to make their assessment of SES. However, the findings of these studies were opposed (Table 1).

### Findings by PIP definition used

The majority of papers assessed PIP through use of the Beers criteria (*n *= 15) [8, 28, 30, 31, 33–36, 38, 39, 41–45]. However, the Beers criteria have been subject to various revisions [23], and different versions were utilised by the different studies included in this review. Additionally, several papers examined only some of the Beers medications, or made other adaptions to the criteria to fit their aims. Overall, nine papers that used some version of the Beers criteria reported a statistically significant association between PIP exposure and deprivation [8, 28, 30, 31, 33, 35, 38, 44, 45]. Of the remaining six, three had mixed findings regarding the association between SES and PIP [36, 39, 43] and a further three reported no significant association [34, 41, 42]. Two studies using the Laroche list [29] and the NBHW criteria [40] found a significant association between SES and PIP, although a Swiss study using the Laroche list found no such association [32]. Finally, a study focussed on the treatment of type 2 diabetes found that patients living in relatively less wealthy countries were significantly more likely to be prescribed a medication with a higher risk of causing hypoglycaemia [27].

## Discussion

### Key Findings

This systematic review suggests that exposure to socioeconomic deprivation has potential to increase older individuals’ likelihood of being exposed to PIP. To our knowledge, this is the first such review to examine this topic. More generally, the review adds to a larger body of evidence linking socioeconomic deprivation and adverse health outcomes [11]. Of the twenty papers analysed, twelve (60%) identified a statistically significant association between SES and PIP [8, 27–31, 33, 35, 38, 40, 44, 45]; in three further studies there appeared to be a significant association in some subgroups but not others [36, 39, 43]. Four studies found no significant association [32, 34, 41, 42], and a single study reported an inverse relationship between SES and PIP [37].

### Interpretation

There are several potential reasons for a link between SES and PIP. Firstly, it has been hypothesised that access to good quality health services is worse amongst patients living in more deprived areas. This may mean that patients living in deprived areas have less access to services such as medication reviews and specialist input regarding their medication regimens [36], which could both help prevent and correct PIP. However, one paper included in this review did not find that access to GPs, nurses and pharmacists was any worse in deprived areas [29]; research from elsewhere has also questioned the so-called ‘inverse care law’ [46]. As such, relative lack of access to healthcare services may not be the reason for PIP’s association with deprivation, although a firm conclusion cannot be drawn based on a single study.

Second, medication costs are another potential factor. In settings where medications need to be paid for, or accessed via health insurance schemes, lack of resources could limit patients’ ability to access ‘gold standard’ medications. It is known that patients’ SES and insurance status can affect clinical decision making [47, 48], and on prescribing choices made [47]. However, it seems unlikely that prescribers would consciously choose to prescribe potentially inappropriate drugs with known issues in the older population for reasons of cost. Furthermore, an association between PIP and SES was observed in settings where medication costs to patients were capped [40], reimbursed [39], or entirely absent [44].

Third, it has been suggested that patients with lower SES take a more passive role in medical consultations, and are less likely to raise concerns with medical practitioners [49]. This may lead to poorer patients being more tolerant of ill-effects associated with inappropriate prescribing, and less likely to request a switch to alternative treatments. Qualitative research has identified a number of reasons as to why older patients comply with potentially inappropriate medications [50], although the potential role of SES does not appear to have been explored.

Finally, the association between deprivation and PIP may be a result of more deprived populations being more likely to experience polypharmacy [51]. Being on more medications increases the risk of medication-related harm, as each additional drug poses a risk of side effects, and each has the potential to interact with other medications being taken [52]. Based on their own findings, one included study [31] remarked that, in the UK at least, ‘deprivation appears to exert its effect on PIP through an increased risk of receiving any drug’, with the apparent association between SES and PIP ‘almost completely’ explained by patients with lower SES being on more medications. However, our review would suggest that this is not the sole explanatory variable. Several included studies identified an association between SES and PIP even after adjusting for some measure of polypharmacy [8, 35, 38, 40, 45].

### Strengths and limitations

This review has several strengths. It identified papers covering a range of countries and settings, and examined several aspects of PIP. Many of the included papers featured study cohorts numbering several thousand, allowing statistically significant results to be obtained. No restrictions were placed on publication language, resulting in one Portuguese-language paper [33] being included as well as nineteen in English.

The majority of included studies used some version of the Beers criteria, allowing a degree of comparability. Conversely, including papers that examined PIP using other approaches added an additional dimension to our findings. Examining inappropriate treatment intensification in type 2 diabetics and PIP using alternative objective criteria (eg Laroche/NBHW classification) allowed the association between PIP and deprivation to be examined from different perspectives other than that provided by Beers. The finding that significant associations between PIP and deprivation were observed from these different perspectives strengthens our conclusions.

The review also has limitations. Deprivation in particular is a heterogenous concept, there is no universally-applied, standard way to assess individuals’ socioeconomic status and, by extension, their experience of deprivation [18]. Our inclusion criteria captured papers explicitly referring to income and wealth-based measures of socioeconomic status in their title or abstract. It is possible that this approach may have been too narrow, and may not have captured papers that determined or referred to deprivation in another way. Similarly, it is possible that some multivariate analyses set out to examine a link between SES and PIP, found no significant association, and decided not to report this finding in their titles/abstracts, particularly if other statistically significant findings were identified. Such papers would not have been identified during literature review, which could have affected the conclusions drawn regarding the association between SES and PIP.

While the review appears to illustrate that socioeconomic deprivation may be associated with PIP, it cannot be stated that this is the case in all settings and at all points in time. For example, over half of Africa’s population lacks access to even essential medications, with poverty playing a major role [53]. Amongst this deprived population it would be surprising to see a high prevalence of PIP, given that access to any sort of medication is limited. Socioeconomic status should have a relatively lower impact in countries with universal healthcare provision. However, important effect modifiers may still complicate access to prescribed medication, and therefore the relationship between SES and PIP. For example, three regions of the UK (Scotland, Wales, Northern Ireland) provide free prescriptions for all NHS patients. In England however, a prescription charge of £9.65 is payable for each item prescribed in primary care, unless the patient qualifies for an exemption from payment [54]. Though the receipt of certain forms of income support may entitle a patient in England to free prescriptions, it may be the case that a degree of differential access to medications exists within the UK. In turn, this may mean that the relationship between SES and PIP may be different in different parts of the country, despite universal healthcare provision. Finally, the relationship between SES and PIP may change in a country over time. To use an example discussed by Odubanjo and colleagues [44], when access to subsidised prescriptions is means-tested according to income, changing this means-testing may have a significant impact on medication access and likelihood of PIP exposure.

Despite suggesting an association between socioeconomic status and PIP, this systematic review cannot make any firm conclusions regarding causality or effect size. While improving the socioeconomic circumstances of older people around the world would almost certainly have a number of benefits, there is no cast iron guarantee that this would have an impact on PIP incidence. Additionally, socioeconomic status is not a readily modifiable attribute, and is one of many factors (both modifiable and non-modifiable) that may play a role in older persons’ likelihood of experiencing PIP. For example, the papers included in this review found that gender, ethnicity, educational attainment and marital status may also have an impact on PIP, among many other factors.

### Implications for practice and policy

Our findings could be used to target strategies for medication review. Such reviews are an important aspect of medical practice [55], but the ability of practitioners to conduct such reviews in real-world settings is limited by time and resource constraints [56]. This review highlights that prioritising older people living in socioeconomically-deprived circumstances may be an efficient strategy when carrying out medication reviews, as is already recommended in some jurisdictions [57].

### Implications for research

Four studies used more than one measure of SES. Three of these found no statistically significant link between PIP and either measure of SES. However, the fourth study [36] found that individuals' household income did not seem to be linked to their odds of being prescribed multiple PIM, although evidence for an association was found when information on individuals' income, education level and neighbourhood were combined to create a 'cumulative' measure of SES. This observation, though not readily explicable, does establish that the way SES is measured is important, and can have an impact on the conclusions made by studies. It may also suggest that using a ‘holistic’ measure of SES based on multiple factors may better identify significant associations between SES and PIP, where they exist. Further research would be needed to test this hypothesis.

## Conclusions

Our findings suggest that there may be an association between an older persons’ SES and their likelihood of being exposed to PIP. This appears to be the case when several different measures of socioeconomic status are used, and when PIP is examined using a range of criteria. Most of the papers included in this study used multivariate analyses, and socioeconomic status appears to be one of several factors associated with an older person’s likelihood of experiencing PIP even when controlling for other factors. This suggests that targeting older people affected by deprivation for medication review may be an important strategy.

### Supplementary Information

Supplementary Material 1.

Supplementary Material 2.

Supplementary Material 3.

## Data Availability

All data generated or analysed during this study are included in this published article [and its supplementary information files].

## Abbreviations

IMD: Index of Multiple Deprivation
JBI: Joanna Briggs Institute
NBHW: Swedish National Board of Health and Welfare classification
PIM: Potentially inappropriate medication
PIP: Potentially inappropriate prescribing
PROSPERO: International Prospective Register of Systematic Reviews
SES: Socioeconomic status
START: Screening tool to alert doctors to right treatments
STOPP: Screening tool of older people’s potentially inappropriate prescriptions
SWiM: Synthesis without meta-analysis

## References

[1] Maresova P, Javanmardi E, Barakovic S, Barakovic Husic J, Tomsone S, Krejcar O, et al. Consequences of chronic diseases and other limitations associated with old age – a scoping review. BMC Public Health. 2019;19(1):1431. 31675997 10.1186/s12889-019-7762-5 PMC6823935

[2] Shenkin SD, Harrison JK, Wilkinson T, Dodds RM, Ioannidis JPA. Systematic reviews: guidance relevant for studies of older people. Age Ageing. 2017;46(5):722–8. 28655142 10.1093/ageing/afx105 PMC5860219

[3] Atella V, Piano Mortari A, Kopinska J, Belotti F, Lapi F, Cricelli C, et al. Trends in age-related disease burden and healthcare utilization. Aging Cell. 2019;18(1):e12861. 30488641 10.1111/acel.12861 PMC6351821

[4] World Health Organisation. WHO Newsroom. 2022 [cited 2023 Sep 11]. Ageing and health. Available from: https://www.who.int/news-room/fact-sheets/detail/ageing-and-health.

[5] Christensen K, Doblhammer G, Rau R, Vaupel JW. Ageing populations: the challenges ahead. Lancet. 2009;374(9696):1196–208. 19801098 10.1016/S0140-6736(09)61460-4 PMC2810516

[6] NICE. NICE Clinical Knowledge Summaries. 2023 [cited 2023 Sep 12]. Multimorbidity. Available from: https://cks.nice.org.uk/topics/multimorbidity/background-information/prevalence/.

[7] Ho ISS, Azcoaga-Lorenzo A, Akbari A, Davies J, Hodgins P, Khunti K, et al. Variation in the estimated prevalence of multimorbidity: systematic review and meta-analysis of 193 international studies. BMJ Open. 2022;12(4):e057017. 35487738 10.1136/bmjopen-2021-057017 PMC9058768

[8] Holmes HM, Luo R, Kuo YF, Baillargeon J, Goodwin JS. Association of Potentially Inappropriate Medicine Use with Patient and Prescriber Characteristics in Medicare Part D. Pharmacoepidemiol Drug Saf. 2013;22(7):728–34. 23494811 10.1002/pds.3431 PMC3701724

[9] Spinewine A, Schmader KE, Barber N, Hughes C, Lapane KL, Swine C, et al. Appropriate prescribing in elderly people: how well can it be measured and optimised? Lancet Lond Engl. 2007;370(9582):173–84. 10.1016/S0140-6736(07)61091-5 17630041

[10] Cahir C, Moriarty F, Teljeur C, Fahey T, Bennett K. Potentially inappropriate prescribing and vulnerability and hospitalization in older community-dwelling patients. Ann Pharmacother. 2014;48(12):1546–54. 25248541 10.1177/1060028014552821

[11] Zhang CQ, Chung PK, Zhang R, Schüz B. Socioeconomic Inequalities in Older Adults’ Health: The Roles of Neighborhood and Individual-Level Psychosocial and Behavioral Resources. Front Public Health. 2019;25(7):318. 10.3389/fpubh.2019.00318 PMC6823619 31709222

[12] Steptoe A, Zaninotto P. Lower socioeconomic status and the acceleration of aging: An outcome-wide analysis. Proc Natl Acad Sci. 2020;117(26):14911–7. 32541023 10.1073/pnas.1915741117 PMC7334539

[13] Foster HME, Celis-Morales CA, Nicholl BI, Petermann-Rocha F, Pell JP, Gill JMR, et al. The effect of socioeconomic deprivation on the association between an extended measurement of unhealthy lifestyle factors and health outcomes: a prospective analysis of the UK Biobank cohort. Lancet Public Health. 2018;3(12):e576–85. 30467019 10.1016/S2468-2667(18)30200-7

[14] Tudor HJ. The Inverse Care Law. The Lancet. 1971;297(7696):405–12. 10.1016/s0140-6736(71)92410-x 4100731

[15] United Nations Refugee Agency. UNHCR. 2020 [cited 2023 Aug 29]. Older persons. Available from: https://emergency.unhcr.org/protection/persons-risk/older-persons.

[16] The OECD. OECD Data. 2023 [cited 2023 Aug 29]. Elderly population: OECD Data. Available from: http://data.oecd.org/pop/elderly-population.htm.

[17] NHS England. england.nhs.uk. 2023 [cited 2023 Aug 29]. NHS England - Improving care for older people. Available from: https://www.england.nhs.uk/ourwork/clinical-policy/older-people/improving-care-for-older-people/.

[18] Galobardes B, Shaw M, Lawlor DA, Lynch JW. Indicators of socioeconomic position (part 1). J Epidemiol Community Health. 2006;60(1):7–12. 16361448 10.1136/jech.2004.023531 PMC2465546

[19] Galobardes B, Shaw M, Lawlor DA, Lynch JW, Davey SG. Indicators of socioeconomic position (part 2). J Epidemiol Community Health. 2006;60(2):95–101. 16415256 10.1136/jech.2004.028092 PMC2566160

[20] Ministry of Housing, Communities and Local Government. The English Indices of Deprivation 2019: Statistical Release [Internet]. 2019. Available from: https://assets.publishing.service.gov.uk/media/5d8e26f6ed915d5570c6cc55/IoD2019_Statistical_Release.pdf.

[21] US Department of Health & Human Services. HHS.gov. 2022 [cited 2023 Aug 29]. What’s the difference between Medicare and Medicaid? Available from: https://www.hhs.gov/answers/medicare-and-medicaid/what-is-the-difference-between-medicare-medicaid/index.html.

[22] O’Mahony D, O’Sullivan D, Byrne S, O’Connor MN, Ryan C, Gallagher P. STOPP/START criteria for potentially inappropriate prescribing in older people: version 2. Age Ageing. 2015;44(2):213–8. 25324330 10.1093/ageing/afu145 PMC4339726

[23] American Geriatrics Society. American Geriatrics Society 2023 updated AGS Beers Criteria for potentially inappropriate medication use in older adults. J Am Geriatr Soc. 2023;71(7):2052–81. 37139824 10.1111/jgs.18372 PMC12478568

[24] Joanna Briggs Institute. Critical Appraisal Tools [Internet]. 2023 [cited 2023 Sep 1]. Available from: https://jbi.global/critical-appraisal-tools.

[25] Page MJ, McKenzie JE, Bossuyt PM, Boutron I, Hoffmann TC, Mulrow CD, et al. The PRISMA 2020 statement: an updated guideline for reporting systematic reviews. BMJ. 2021;29(372):n71. 10.1136/bmj.n71 PMC8005924 33782057

[26] Campbell M, McKenzie JE, Sowden A, Katikireddi SV, Brennan SE, Ellis S, et al. Synthesis without meta-analysis (SWiM) in systematic reviews: reporting guideline. BMJ. 2020;16:l6890. 10.1136/bmj.l6890 PMC7190266 31948937

[27] Bongaerts B, Arnold SV, Charbonnel BH, Chen H, Cooper A, Fenici P, et al. Inappropriate intensification of glucose-lowering treatment in older patients with type 2 diabetes: the global DISCOVER study. BMJ Open Diabetes Res Care. 2021;9(1):e001585. 33941550 10.1136/bmjdrc-2020-001585 PMC8098925

[28] Abraham DS, Pham Nguyen TP, Hennessy S, Weintraub D, Gray SL, Xie D, et al. Frequency of and risk factors for potentially inappropriate medication use in Parkinson’s disease. Age Ageing. 2020;49(5):786–92. 32255485 10.1093/ageing/afaa033 PMC7444670

[29] Beuscart JB, Genin M, Dupont C, Verloop D, Duhamel A, Defebvre MM, et al. Potentially inappropriate medication prescribing is associated with socioeconomic factors: a spatial analysis in the French Nord-Pas-de-Calais Region. Age Ageing. 2017;46(4):607–13. 28064169 10.1093/ageing/afw245

[30] Blackwell SA, Montgomery MA, Baugh DK, Ciborowski GM, Riley GF. Applying the 2003 Beers Update to Elderly Medicare Enrollees in the Part D Program. Medicare Medicaid Res Rev. 2012;2(2):mmrr.002.02.a01. 24800144 10.5600/mmrr.002.02.a01 PMC4006423

[31] Carey IM, De Wilde S, Harris T, Victor C, Richards N, Hilton SR, et al. What Factors Predict Potentially Inappropriate Primary Care Prescribing in Older People? Drugs Aging. 2008;25(8):693–706. 18665661 10.2165/00002512-200825080-00006

[32] Chauvin P, Fustinoni S, Seematter-Bagnoud L, Herr M, Santos EB. Potentially inappropriate prescriptions: Associations with the health insurance contract and the quality of the patient–physician relationship? Health Policy. 2021;125(9):1146–57. 34266705 10.1016/j.healthpol.2021.06.011

[33] Coelho Filho JM, Marcopito LF, Castelo A. Medication use patterns among elderly people in urban area in Northeastern Brazil. Rev Saude Publica. 2004;38(4):557–64. 15311297 10.1590/s0034-89102004000400012

[34] Extavour RM, Perri M. Patient, Physician, and Health-System Factors Influencing the Quality of Antidepressant and Sedative Prescribing for Older. Community-Dwelling Adults Health Serv Res. 2018;53(1):405–29. 28024315 10.1111/1475-6773.12641 PMC5785327

[35] Fialova D, Topinkova E, Gambassi G, Finne-Soveri H, Jonsson PV, Carpenter I, et al. Potentially inappropriate medication use among elderly home care patients in Europe. JAMA. 2005;293(11):1348–58. 15769968 10.1001/jama.293.11.1348

[36] Hwang J, Lyu B, Ballew S, Coresh J, Grams ME, Couper D, et al. The association between socioeconomic status and use of potentially inappropriate medications in older adults. J Am Geriatr Soc. 2023;71(4):1156–66. 36511705 10.1111/jgs.18165 PMC10089965

[37] Hyttinen V, Jyrkkä J, Saastamoinen LK, Vartiainen AK, Valtonen H. Patient- and health care-related factors associated with initiation of potentially inappropriate medication in community-dwelling older persons. Basic Clin Pharmacol Toxicol. 2019;124(1):74–83. 30003664 10.1111/bcpt.13096

[38] Joung KI, Shin JY, Cho SI. Features of anticholinergic prescriptions and predictors of high use in the elderly: Population-based study. Pharmacoepidemiol Drug Saf. 2019;28(12):1591–600. 31692168 10.1002/pds.4902

[39] Lechevallier-Michel N, Gautier-Bertrand M, Alperovitch A, Berr C, Belmin J, Legrain S, et al. Frequency and risk factors of potentially inappropriate medication use in a community-dwelling elderly population: results from the 3C Study. Eur J Clin Pharmacol. 2005;60(11):813–9. 15599504 10.1007/s00228-004-0851-z

[40] Lesen E, Andersson K, Petzold M, Carlsten A. Socioeconomic determinants of psychotropic drug utilisation among elderly: a national population-based cross-sectional study. BMC Public Health. 2010;10(100968562):118. 20214796 10.1186/1471-2458-10-118 PMC2845562

[41] Lutz BH, Miranda VIA, Bertoldi AD. Potentially inappropriate medications among older adults in Pelotas, Southern Brazil. Rev Saúde Pública. 2017;13(51):52. 10.1590/S1518-8787.2017051006556 PMC5493363 28658367

[42] Miller GE, Sarpong EM, Davidoff AJ, Yang EY, Brandt NJ, Fick DM. Determinants of Potentially Inappropriate Medication Use among Community-Dwelling Older Adults. Health Serv Res. 2017;52(4):1534–49. 27686781 10.1111/1475-6773.12562 PMC5517671

[43] Morgan SG, Weymann D, Pratt B, Smolina K, Gladstone EJ, Raymond C, et al. Sex differences in the risk of receiving potentially inappropriate prescriptions among older adults. Age Ageing. 2016;45(4):535–42. 27151390 10.1093/ageing/afw074 PMC4916346

[44] Odubanjo E, Bennett K, Feely J. Influence of socioeconomic status on the quality of prescribing in the elderly – a population based study. Br J Clin Pharmacol. 2004;58(5):496–502. 15521897 10.1111/j.1365-2125.2004.02179.x PMC1884614

[45] Rahman M, Howard G, Qian J, Garza K, Abebe A, Hansen R. Disparities in the appropriateness of medication use: Analysis of the REasons for Geographic And Racial Differences in Stroke (REGARDS) population-based cohort study. Res Soc Adm Pharm RSAP. 2020;16(12):1702–10. 10.1016/j.sapharm.2020.02.008 PMC7438264 32098707

[46] Barlow P, Mohan G, Nolan A, Lyons S. Area-level deprivation and geographic factors influencing utilisation of General Practitioner services. SSM - Popul Health. 2021;11(15):100870. 10.1016/j.ssmph.2021.100870 PMC8342788 34386571

[47] Bernheim SM, Ross JS, Krumholz HM, Bradley EH. Influence of Patients’ Socioeconomic Status on Clinical Management Decisions: A Qualitative Study. Ann Fam Med. 2008;6(1):53–9. 18195315 10.1370/afm.749 PMC2203396

[48] Meyers DS, Mishori R, McCann J, Delgado J, O’Malley AS, Fryer E. Primary Care Physicians’ Perceptions of the Effect of Insurance Status on Clinical Decision Making. Ann Fam Med. 2006;4(5):399–402. 17003138 10.1370/afm.574 PMC1578641

[49] Allen S, Rogers SN, Brown S, Harris RV. What are the underlying reasons behind socioeconomic differences in doctor-patient communication in head and neck oncology review clinics? Health Expect Int J Public Particip Health Care Health Policy. 2021;24(1):140–51. 10.1111/hex.13163 PMC7879543 33227177

[50] Heser K, Pohontsch NJ, Scherer M, Löffler A, Luck T, Riedel-Heller SG, et al. Perspective of elderly patients on chronic use of potentially inappropriate medication – Results of the qualitative CIM-TRIAD study. PLoS ONE. 2018;13(9):e0202068. 30231027 10.1371/journal.pone.0202068 PMC6145513

[51] Iqbal A, Richardson C, Iqbal Z, O’Keefe H, Hanratty B, Matthews FE, et al. Are there socioeconomic inequalities in polypharmacy among older people? A systematic review and meta-analysis. BMC Geriatr. 2023;23(1):149. 36934249 10.1186/s12877-023-03835-z PMC10024437

[52] Varghese D, Ishida C, Haseer Koya H. Polypharmacy [Internet]. Treasure Island (FL): StatPearls Publishing; 2023 [cited 2023 Sep 25]. Available from: http://www.ncbi.nlm.nih.gov/books/NBK532953/.

[53] Yenet A, Nibret G, Tegegne BA. Challenges to the Availability and Affordability of Essential Medicines in African Countries: A Scoping Review. Clinicoecon Outcomes Res. 2023;13(15):443–58. 10.2147/CEOR.S413546 PMC10276598 37332489

[54] Waitzman E. House of Lords Library. 2023 [cited 2024 Feb 13]. Free NHS prescriptions: Eligibility for benefit claimants. Available from: https://lordslibrary.parliament.uk/free-nhs-prescriptions-eligibility-for-benefit-claimants/.

[55] Shepherd AB. The importance of medication reviews in a primary care setting. Nurse Prescr. 2018;16(6):280–7.

[56] Duncan P, Cabral C, McCahon D, Guthrie B, Ridd MJ. Efficiency versus thoroughness in medication review: a qualitative interview study in UK primary care. Br J Gen Pract. 2019;69(680):e190–8. 30745357 10.3399/bjgp19X701321 PMC6400610

[57] Mair A, Wilson M, Dreischulte T. The polypharmacy programme in Scotland: realistic prescribing. Prescriber. 2019;30(8):10–6.

