# Supplementary material for: Potentially inappropriate prescribing (PIP) in older people and its association with socioeconomic deprivation—a systematic review and narrative synthesis
Source: BMC Geriatr. 2024 Aug 2;24:651. doi: 10.1186/s12877-024-04858-w (PMC11295679; doi:10.1186/s12877-024-04858-w)
Supplement: Supplementary file 1 — Supplementary Material 1. [file 12877_2024_4858_MOESM1_ESM.docx]

Appendix 1: Data extraction table for included studies

| First Author & Year | Location | Setting & Participants | Socioeconomic Status (SES) Indicator(s) | Potentially Inappropriate Prescribing (PIP) Measure(s) | Relevant Data Extracted Regarding Association Between SES & PIP |
| --- | --- | --- | --- | --- | --- |
| Abraham 2020(1) | USA | 458,086 Medicare beneficiaries who were at least 65 years old and had Medicare part A (inpatient), B (outpatient), and D (prescription) coverage for the full 2014 calendar year, along with a minimum of one prescription claim and a diagnosis of Parkinson’s disease (PD) | Medicaid eligibility | Beers criteria (2015) | Medicaid eligibility was significantly associated with a higher risk of potentially inappropriate medication use in Parkinson's disease patients  Adjusted odds ratio (aOR) for any potentially inappropriate medication (PIM) use in Medicaid-eligible beneficiaries was 1.16 (95% Confidence Interval/CI 1.15-1.18) compared to non-Medicaid-eligible beneficiaries  aOR for use of a PIM with the potential to worsen PD motor symptoms was 2.42 (95% CI 2.37-2.45) among Medicaid-eligible beneficiaries compared to non-Medicaid-eligible beneficiaries  aOR for use of a PIM with the potential to worsen cognitive impairment was 1.25 (95% CI 1.23-1.26) among Medicaid-eligible beneficiaries compared to non-Medicaid-eligible beneficiaries |
| Beuscart 2017(2) | France  (Nord-Pas-de-Calais) | 207,979 people aged 75 years and over living in the Nord-Pas-de-Calais who had a medication dispensed by a community pharmacy between 1 January and 31 March 2012 | Municipality average taxable income, non-taxable income | Laroche list | A statistically significant association was reported between income and PIP  Municipalities with a high prevalence of PIP had a lower median taxable income than areas with a low prevalence of PIP (p < 0.0001)  Municipalities with a high prevalence of PIP had a lower median non-taxable income than areas with a low prevalence of PIP (p < 0.0001)  Municipalities classified as having neither a high or low prevalence of PIP had a median taxable income significantly higher than high PIP areas, but significantly lower than low PIP areas (p < 0.0001) |
| Blackwell 2012(3) | USA | Medicare Part D enrolees aged 65 or older, and who had received at least one prescription drug during the year 2007 (n=16,554,524) | Eligibility for both Medicare and Medicaid coverage (‘dual enrolees’) | Beers criteria (2003) | The likelihood of Beers drug use among dual enrolees was significantly higher than among non-duals (aOR 1.023, 95% CI 1.020–1.026; p < 0.0001) |
| Bongaerts 2021(4) | Algeria, Argen- tina, Australia, Austria, Bahrain, Brazil, Canada,  Colombia, Costa Rica, Czech Republic, Denmark, Egypt, France, India, Indonesia, Italy, Japan, Jordan, Kuwait, Lebanon, Malaysia, Mexico, the Netherlands, Norway, Oman, Panama, Poland, Russia, Saudi Arabia, South Africa, South Korea, Spain, Sweden, Taiwan, Tunisia, Turkey, United Arab Emirates | Patients with type 2 diabetes (aged ≥65 years) who had initiated second-line glucose-lowering therapy between January 2014 and December 2016 (n=3344) | Gross national income (GNI) per capita of patients’ country of residence | HbA1c level indicative of inappropriately intensive glycaemic control [<7.0% (53.0 mmol/mol)], or inappropriate initiation of a high-risk glucose-lowering medication (e.g., sulfonylureas, meglitinides, or insulin) in patients with an HbA1c level <7.0% (53.0 mmol/mol) | Patients from lower-middle and upper-middle income countries had greater odds of receiving high-risk glucose-lowering medication, but overly intensive glycaemic control was more common in high GNI countries  Higher gross national income (per US$5000 increment) was associated with a decreased likelihood of receiving high-risk glucose-lowering medication (OR 0.86, 95% CI = 0.78-0.95, p < 0.05)  Higher gross national income (per US$5000 increment) was associated with a slightly increased likelihood of having a HbA1c indicative of inappropriately intensive glycaemic control (Odds ratio/OR 1.05, 95% CI = 1.00-1.09, p < 0.05) |
| Carey 2008(5) | UK | UK primary care patients aged 65 and older who were prescribed at least one medication in 2005 (n=218,567) | Index of Multiple Deprivation (IMD), Acorn index* | Beers criteria 2003, focusing on inappropriate analgesics, antidepressants and sedative/anxiolytics | Persons in the most deprived IMD quintile (1) had significantly higher odds of experiencing PIP relative the persons in the least deprived IMD quintile (5) (aOR 1.09, 95% CI 1.04-1.15). No significant difference observed between the least deprived quintile and quintiles 2,3 and 4  Compared to wealthy achievers, those in the classes urban prosperity, moderate means and hard pressed all had significantly higher odds of experiencing PIP after adjusting for other factors. For the comfortably off, the odds of experiencing PIP compared to wealthy achievers was higher, but this difference was of borderline statistical significance (aOR 1.03, 95% CI 1.00-1.05)  When the use of inappropriate analgesics and inappropriate antidepressants was examined, those classed has hard pressed had higher odds of experiencing PIP relative to wealthy achievers. Conversely, inappropriate anxiolytic and sedative prescribing was less likely in hard pressed, moderate means and comfortably off groups |
| Chauvin 2021(6) | Switzerland (Vaud canton) | Community-dwelling individuals aged 68 and older living in the canton of Vaud, Switzerland who were taking at least one prescription drug (n=1595) | Receipt of income-related social support, self-reported assets higher than contemporaries | Laroche list | Individuals exposed to PIP were more likely to be in receipt of income-related social support, and less likely to report having assets higher than their contemporaries but these associations did not reach statistical significance after adjusting for other factors |
| CoelFilho 2004(7) | Brazil  (Fortaleza) | 668 individuals aged 60 years or older residing in the city of Fortaleza, Brazil | Area socioeconomic status, derived from Brazilian census statistics | Beers criteria (version unclear) | Statistically significant inverse association observed between higher socioeconomic status and potentially inappropriate prescribing (OR=0.7, 95% CI=0.5-0.9) |
| Extavour 2018(8) | USA | National Ambulatory Medical Care Survey (NAMCS) data on visits to community practices and health centers by patients aged 65+ (number of visits presented, not clear how many individuals were associated with these visits) | Median household income by zip code, Medicaid eligibility | Beers criteria (2012/2015) | Multivariate analysis found no consistent association between median household income quartile, or Medicaid eligibility, and the inappropriate prescribing of antidepressants or sedatives |
| Fialova 2005(9) | Czech Republic, Denmark, Finland, Iceland, Italy,  the Netherlands, Norway, the United Kingdom. | Patients aged 65 and over who received home care services in eight European countries (n=2707) | Self-reported economic situation^ | Criteria drawn up by an expert panel, with reference to Beers criteria (1997, 2003) and Mcleod criteria (1997) | Individuals reporting a poor economic situation had a 1.96-fold higher relative risk of receiving an inappropriate medication than the reference group  After adjusting for other factors, poor economic situation was associated with a significantly higher likelihood of experiencing PIP - aOR 2.48 (95% CI, 1.82-3.39; P < 0.001) |
| Holmes 2013(10) | USA  (Texas) | Medicare Part D enrolees aged 66+ who received at least one prescription drug in 2008 (n=677,580) | Eligibility for low income subsidy | Beers criteria (2003) | Enrolees eligible for a low income subsidy were significantly more likely to receive potentially inappropriate medications (aOR 1.03, 95% CI 1.02-1.05) |
| Hwang 2023(11) | USA  (Washington County, Maryland; Forsyth County, North Carolina; Jackson, Mississippi; Minneapolis, Minnesota) | Community-dwelling adults aged 66-90 years who were enrolled in the Atherosclerosis Risk in Communities Study (ARiC) study (n=4927) and who were taking at least one medication at visit 5 of the study | Neighbourhood SES: based on Area deprivation index (ADI); Individual-level SES: based on household income; and cumulative SES score (index based upon a combination of neighbourhood/individual-level SES measures) | Beers criteria (2019) | Neighbourhood SES: Patients living in the neighbourhoods with the highest ADI (equivalent to the lowest SES) were significantly more likely to be taking two or more PIM than those living in the lowest ADI neighbourhoods (aOR 1.83, 95% CI 1.10-3.05)  Individual-level SES: No significant association observed between income and exposure to two or more PIM  Cumulative SES: After adjusting for other factors, patients with a lower cumulative SES score had higher odds of being on two or more PIMs than patients with a high cumulative SES score – aOR 1.66 (95% CI 1.02-2.71) |
| Hyttinen  2018(12) | Finland | 28,497 community‐dwelling older persons aged ≥65 years with no PIM exposure in the 24 months prior to study and who were followed between 2002 and 2013 | Household income (spending money per head, year 2000) | Meds75+ database classification | Higher income was associated with a significantly higher probability of PIM initiation in persons aged 65‐74 years, with the risk of PIM exposure increasing in accordance with income band  In persons aged ≥75 years there was a less clear association between income and PIM initiation; the risk of PIP among those earning less than €9999 was not significantly different to the risk among those earning >€30,000 |
| Joung 2019(13) | South Korea | 388,629 subjects aged ≥70 years recruited from the 2012 National Health Insurance Service Elderly (NHISE) cohort database | Income | Beers criteria (2015) | High usage of strong anticholinergics was significantly less common among individuals in the top income decile, as compared to individuals in the eighth, fourth or first income decile  The aOR for high use of strong anticholinergic agents was 1.29 (95% CI 1.25-1.33) for the lowest income decile compared to the highest income decile |
| Lechevallier-Michel  2005(14) | France (Bordeaux, Dijon & Montpellier) | 9294 community-dwelling individuals aged 65 years and older who were living in three French towns (Bordeaux, Dijon, and Montpellier) or their suburbs and who were participants in the 3C study | Household income | Criteria drawn up by an expert panel, with reference to Beers criteria (version unclear) | The highest proportion of PIM users (56.5%) was found among those with the lowest incomes (those supported by the National Solidarity Fund)  Low household income was associated with an increased likelihood of PIM usage, but this association did not reach statistical significance in patients taking ≤ 4 drugs |
| Lesen 2010(15) | Sweden | All individuals aged 75 years and older on 1 January 2006 in Sweden who had purchased a prescribed psychotropic drug in 2006 via a community pharmacy (n = 384,712) | Family disposable income in 2005, adjusted for family size | National Board of Health and Welfare's (NBHW) classification of PIP | The probability of utilizing potentially inappropriate psychotropics was higher among individuals with low income than those with high income (aOR 1.14, 95% CI 1.12-1.16)  The use of potentially inappropriate combinations of psychotropics was also more likely among those with a low income, as compared to those with a high income (aOR 1.11, 95% CI 1.08-1.14) |
| Lutz 2017(16) | Brazil  (Pelotas) | 1451 community-dwelling older adults aged 60 years or older who were residents of the urban area of Pelotas, Southern Brazil | Brazilian Association of Research Companies (ABEP) classification of economic level | Beers criteria (2012) | No significant difference in PIP prevalence between individuals in the lowest socioeconomic class (class D/E) and those in the highest socioeconomic class (class A/B) (adjusted prevalence ratio 0.99, 95% CI 0.80-1.23) |
| Miller 2017(17) | USA | Community-dwelling older adults aged 65 and older who acquired at least one prescription medication during the period studied by the Medical Expenditure Panel Survey (2006-2010)  (data presented as occurrences per person-year of data, not clear how many individuals were included) | Income as a percentage of federal poverty level, Medicaid eligibility | Beers criteria (2012) | After adjusting for other factors the study did not find a statistically significant association between income and PIP, or between Medicaid eligibility and PIP |
| Morgan 2016(18) | Canada  (British Columbia) | Residents of British Columbia who were aged 65 and older in 2013 and who were covered by British Columbia's universal, public health insurance program for medical and hospital care, and who were eligible for coverage under British Columbia's universal, public drug benefit plan (n= 660,679) | Household-specific income data (available for 78% of population) and neighbourhood-based proxy incomes (for the other 22%) | Beers criteria (2012) | PIP was most prevalent amongst individuals in the lowest income quintile  After adjusting for other factors, there was a statistically significant association between income and PIP in men but not in women |
| Odubanjo 2004(19) | Ireland  (Eastern region) | General Medical Services (GMS) prescription data for all patients aged 70+ living in the Eastern region of Ireland between July 2001 & December 2002 (n=95,055) | Eligibility for enrolment in the GMS scheme for free medical and pharmaceutical services (more affluent people became eligible for enrolment in the scheme from July 2001) | List compiled by authors following a review of the literature on good and bad prescribing practice in the elderly | Deprivation was associated with a higher likelihood of receiving potentially harmful drugs and drug combinations (OR 1.27, 95% CI 1.24-1.31)  Individuals in the relatively deprived cohort were significantly (p < 0.001) more likely to be prescribed potentially harmful drugs such as cerebral vasodilators (aOR 1.52, 95% CI 1.38-1.69), long-acting sulphonylureas (aOR 1.43, 95% CI 1.20-1.72) and long-acting benzodiazepines (aOR 1.17, 95% CI 1.12-1.21)  Individuals in the relatively deprived cohort were significantly (p < 0.01) more likely to be prescribed potentially inappropriate drug combinations, such as NSAIDs & warfarin (aOR 1.15, 95% CI 1.03-1.28) or NSAIDs & diuretics (aOR 1.39, 95% CI 1.27-1.45)  With regard to evidence-based prescribing, the relatively deprived cohort with IHD was significantly (p < 0.001) less likely to receive secondary prevention therapies such as statins (OR 0.82, 95% CI 0.74-0.90) and beta blockers (OR 0.85, 95% CI 0.77-0.93) |
| Rahman 2020(20) | USA | 13,623 participants aged ≥65 who were enrolled in the REasons for Geographic And Racial Differences in Stroke (REGARDS) study between 2003-2007 | Income | Beers criteria (2015) | Significant differences in income (p = 0.01) were observed between prescribed PIM users and non-users, with a higher proportion of PIM users having an income less than $20,000 per annum  Individuals with a lower income had significantly higher odds of being prescribed a PIM  Compared to those with an annual income ≥$75,000, prescribed PIM use was significantly more likely among those earning <$20,000 (aOR 1.26 (95% CI 1.01-1.57), and those earning between $20,000-$34,999 (aOR 1.24 (95% CI 1.02-1.51) |
| *ACORN index classes in order of increasing deprivation: wealthy achievers, urban prosperity, comfortably off, moderate means, hard pressed  ^ Patients were also asked if they had experienced economic difficulties in the prior 30 days that precluded them from being able to pay for prescribed medications, heating, medical care, adequate nutrition, and home help or home care. Patients reporting any difficulties were classified as having poor economic status | | | | | |

1. Abraham DS, Pham Nguyen TP, Hennessy S, Weintraub D, Gray SL, Xie D, et al. Frequency of and risk factors for potentially inappropriate medication use in Parkinson’s disease. Age Ageing. 2020 Aug 24;49(5):786–92.

2. Beuscart JB, Genin M, Dupont C, Verloop D, Duhamel A, Defebvre MM, et al. Potentially inappropriate medication prescribing is associated with socioeconomic factors: a spatial analysis in the French Nord-Pas-de-Calais Region. Age Ageing. 2017;46(4):607–13.

3. Blackwell SA, Montgomery MA, Baugh DK, Ciborowski GM, Riley GF. Applying the 2003 Beers Update to Elderly Medicare Enrollees in the Part D Program. Medicare Medicaid Res Rev. 2012 May 31;2(2):mmrr.002.02.a01.

4. Bongaerts B, Arnold SV, Charbonnel BH, Chen H, Cooper A, Fenici P, et al. Inappropriate intensification of glucose-lowering treatment in older patients with type 2 diabetes: the global DISCOVER study. BMJ Open Diabetes Res Care. 2021 May;9(1):e001585.

5. Carey IM, De Wilde S, Harris T, Victor C, Richards N, Hilton SR, et al. What Factors Predict Potentially Inappropriate Primary Care Prescribing in Older People? Drugs Aging. 2008 Aug 1;25(8):693–706.

6. Chauvin P, Fustinoni S, Seematter-Bagnoud L, Herr M, Santos Eggimann B. Potentially inappropriate prescriptions: Associations with the health insurance contract and the quality of the patient–physician relationship? Health Policy. 2021 Sep 1;125(9):1146–57.

7. Coelho Filho JM, Marcopito LF, Castelo A. [Medication use patterns among elderly people in urban area in Northeastern Brazil]. Rev Saude Publica. 2004;38(4):557–64.

8. Extavour RM, Perri M. Patient, Physician, and Health‐System Factors Influencing the Quality of Antidepressant and Sedative Prescribing for Older, Community‐Dwelling Adults. Health Serv Res. 2018 Feb;53(1):405–29.

9. Fialova D, Topinkova E, Gambassi G, Finne-Soveri H, Jonsson PV, Carpenter I, et al. Potentially inappropriate medication use among elderly home care patients in Europe. JAMA. 2005;293(11):1348–58.

10. Holmes HM, Luo R, Kuo YF, Baillargeon J, Goodwin JS. Association of Potentially Inappropriate Medicine Use with Patient and Prescriber Characteristics in Medicare Part D. Pharmacoepidemiol Drug Saf. 2013 Jul;22(7):728–34.

11. Hwang J, Lyu B, Ballew S, Coresh J, Grams ME, Couper D, et al. The association between socioeconomic status and use of potentially inappropriate medications in older adults. J Am Geriatr Soc. 2023;71(4):1156–66.

12. Hyttinen V, Jyrkkä J, Saastamoinen LK, Vartiainen AK, Valtonen H. The association of potentially inappropriate medication use on health outcomes and hospital costs in community-dwelling older persons: a longitudinal 12-year study. Eur J Health Econ. 2018;20(2):233–43.

13. Joung K in, Shin JY, Cho S il. Features of anticholinergic prescriptions and predictors of high use in the elderly: Population-based study. Pharmacoepidemiol Drug Saf. 2019;28(12):1591–600.

14. Lechevallier-Michel N, Gautier-Bertrand M, Alpérovitch A, Berr C, Belmin J, Legrain S, et al. Frequency and risk factors of potentially inappropriate medication use in a community-dwelling elderly population: results from the 3C Study. Eur J Clin Pharmacol. 2005 Jan;60(11):813–9.

15. Lesén E, Andersson K, Petzold M, Carlsten A. Socioeconomic determinants of psychotropic drug utilisation among elderly: a national population-based cross-sectional study. BMC Public Health. 2010 Mar 9;10(1):118.

16. Lutz BH, Miranda VIA, Bertoldi AD. Potentially inappropriate medications among older adults in Pelotas, Southern Brazil. Rev Saúde Pública. 2017 Jun 13;51:52.

17. Miller GE, Sarpong EM, Davidoff AJ, Yang EY, Brandt NJ, Fick DM. Determinants of Potentially Inappropriate Medication Use among Community‐Dwelling Older Adults. Health Serv Res. 2017 Aug;52(4):1534–49.

18. Morgan SG, Weymann D, Pratt B, Smolina K, Gladstone EJ, Raymond C, et al. Sex differences in the risk of receiving potentially inappropriate prescriptions among older adults. Age Ageing. 2016;45(4):535–42.

19. Odubanjo E, Bennett K, Feely J. Influence of socioeconomic status on the quality of prescribing in the elderly -- a population based study. Br J Clin Pharmacol. 2004 Nov;58(5):496–502.

20. Rahman M, Howard G, Qian J, Garza K, Abebe A, Hansen R. Disparities in the appropriateness of medication use: Analysis of the REasons for Geographic And Racial Differences in Stroke (REGARDS) population-based cohort study. Res Soc Adm Pharm RSAP. 2020 Dec;16(12):1702–10.
