# Supplementary material for: Potentially inappropriate prescribing (PIP) in older people and its association with socioeconomic deprivation—a systematic review and narrative synthesis
Source: BMC Geriatr. 2024 Aug 2;24:651. doi: 10.1186/s12877-024-04858-w (PMC11295679; doi:10.1186/s12877-024-04858-w)
Supplement: Supplementary file 2 — Supplementary Material 2. [file 12877_2024_4858_MOESM2_ESM.docx]

Search Strategies

**Medline (Ovid)**

1 exp Socioeconomic Factors/

2 Healthcare Disparities/

3 exp health inequities/

4 ((Social or class or economic* or Socioeconomic or societal) adj2 (inequalit* or inequit* or disparit* or equit* or disadvantage* or gradient* or differ* or status)).mp.

5 ((healthcare or health) adj2 (inequalit* or inequit* or disparit* or equit* or disadvantage* or prejudice*)).mp.

6 1 or 2 or 3 or 4

7 Inappropriate Prescribing/

8 ((Prescrib* or drug* or Medication) adj2 (Inappropriate or quality or omission* or Suboptimal)).mp.

9 (Under presc* or Underpresc*).mp.

10 (Over presc* or Overpresc*).mp.

11 (STOPP adj START).mp.

12 STOPP.mp.

13 Beers criteria.mp.

14 ((PRISCUS or PIM) adj list*).mp.

15 7 or 8 or 9 or 10 or 11 or 12 or 13 or 14

16 6 and 15

17 limit 16 to yr="2000 -Current"

**Embase (Ovid)**

1 exp Socioeconomic Factors/

2 Healthcare Disparities/

3 exp health inequities/

4 ((Social or class or economic* or Socioeconomic or societal) adj2 (inequalit* or inequit* or disparit* or equit* or disadvantage* or gradient* or differ* or status)).mp.

5 ((healthcare or health) adj2 (inequalit* or inequit* or disparit* or equit* or disadvantage* or prejudice*)).mp.

6 1 or 2 or 3 or 4

7 Inappropriate Prescribing/

8 ((Prescrib* or drug* or Medication) adj2 (Inappropriate or quality or omission* or Suboptimal)).mp.

9 (Under presc* or Underpresc*).mp.

10 (Over presc* or Overpresc*).mp.

11 (STOPP adj START).mp.

12 STOPP.mp.

13 Beers criteria.mp.

14 ((PRISCUS or PIM) adj list*).mp.

15 7 or 8 or 9 or 10 or 11 or 12 or 13 or 14

16 6 and 15

17 limit 16 to yr="2000 -Current"

**CINAHL (Ebsco)**

1 MH “Socioeconomic Factors”

2 MH “Healthcare Disparities”

3 MH “health inequities”

4 TI (Social or class or economic* or Socioeconomic or societal) N1 (inequalit* or inequit* or disparit* or equit* or disadvantage* or gradient* or differ* or status) or AB (Social or class or economic* or Socioeconomic or societal) N1 (inequalit* or inequit* or disparit* or equit* or disadvantage* or gradient* or differ* or status)

5 TI (healthcare or health) N1 (inequalit* or inequit* or disparit* or equit* or disadvantage* or prejudice*) or AB (healthcare or health) N1 (inequalit* or inequit* or disparit* or equit* or disadvantage* or prejudice*)

6 S1 or S2 or S3 or S4

7 MH “Inappropriate Prescribing”

8 TI (Prescrib* or drug* or Medication) N1 (Inappropriate or quality or omission* or Suboptimal) or AB (Prescrib* or drug* or Medication) N1 (Inappropriate or quality or omission* or Suboptimal)

9 TI (Under presc* or Underpresc*) or AB (Under presc* or Underpresc*)

10 TI (Over presc* or Overpresc*) or AB (Over presc* or Overpresc*)

11 TI (STOPP N1 START) or AB (STOPP N1 START)

12 TI STOPP or AB STOPP

13 T1 (Beers criteria) or AB Beers (criteria)

14 TI ((PRISCUS or PIM) N1 list*) or AB ((PRISCUS or PIM) N1 list*)

15 S7 or S8 or S9 or S10 or S11 or S12 or S13 or S14

16 S6 and S15

17 S16 (Limiters – Published Date 20000101-)
